# Supplementary figures and images for: Personalized tumor-specific DNA junctions to detect circulating tumor in patients with endometrial cancer
Source: PLoS One. 2021 Jun 10;16(6):e0252390. doi: 10.1371/journal.pone.0252390 (PMC8192008; doi:10.1371/journal.pone.0252390)

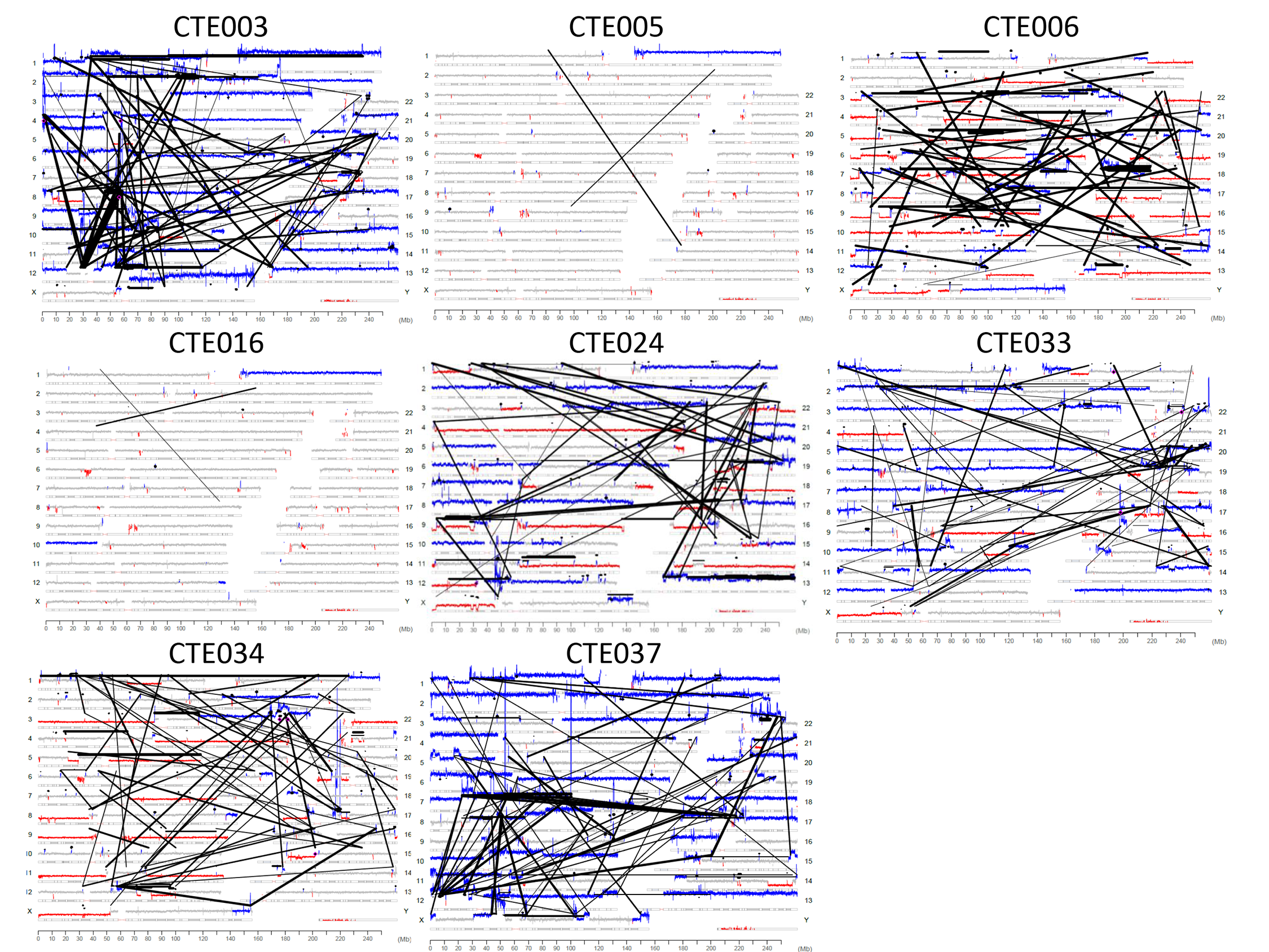

Supplement: S1 Fig — Chromosomes are listed on the left and right Y-axis’; basepair position is on the X axis. Grey cytobands indicate genomic loci bands. The height of the dots each represents the average number of reads over 30 k bases. Grey color indicates wild-type 2 copy state of DNA, blue indicates gains, red indicates losses. Black dots indicate small intrachromosomal rearrangements, while the black lines indicate interchromosomal rearrangements or larger intrachromosomal junctions. (TIF) [file pone.0252390.s001.tif]

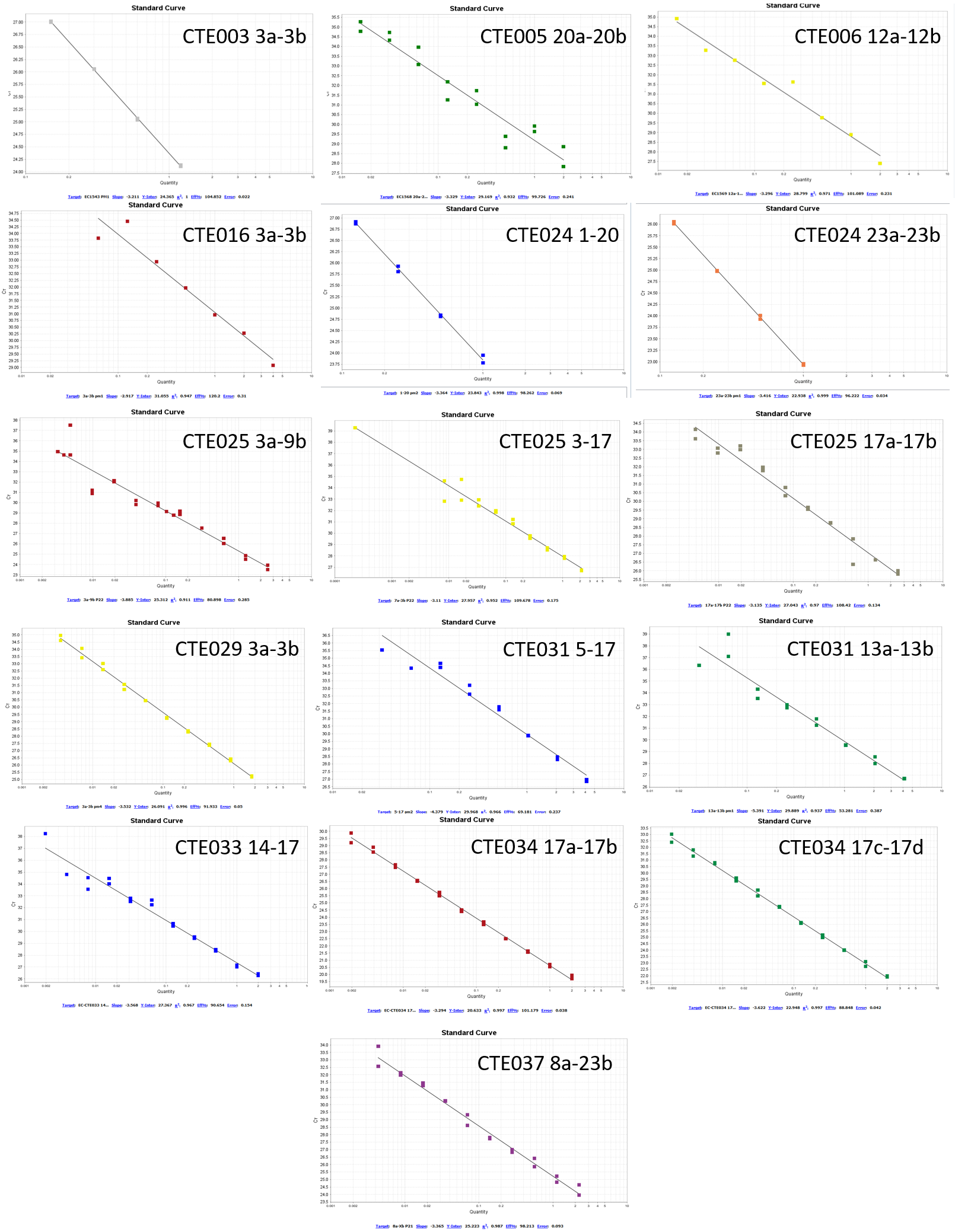

Supplement: S2 Fig — qPCR standard curves produced by Applied Biosystems ViiA-7 generated for each junctions primer pair tested in cfDNA. Y-axis: Ct, X-axis quantity of input (ng/ul). (TIF) [file pone.0252390.s002.tif]

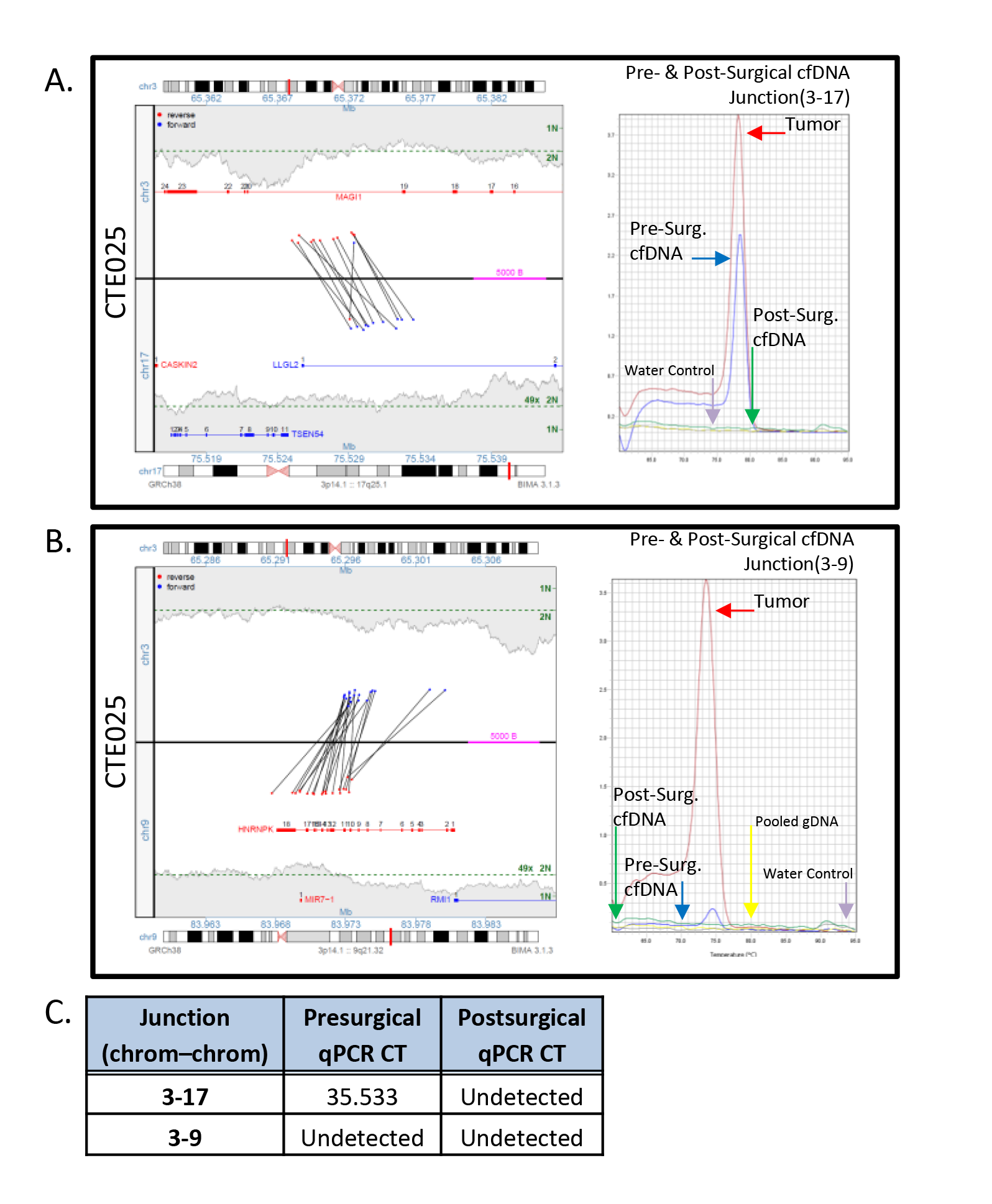

Supplement: S3 Fig — (A) Left Panel: Junction plot of selected junction. Right Panel: Sybr green qPCR melting curve of amplicons of 3–17 junction in patient pre- and post-surgical cfDNA. (B) (A) Left Panel: Junction plot of selected junction. Right Panel: Sybr green qPCR melting curve of amplicons of 3–9 junction in patient pre- and post-surgical cfDNA. (C) qPCR CT values for each junction (TIF) [file pone.0252390.s003.tif]
