# Supplementary material for: Personalized tumor-specific DNA junctions to detect circulating tumor in patients with endometrial cancer
Source: PLoS One. 2021 Jun 10;16(6):e0252390. doi: 10.1371/journal.pone.0252390 (PMC8192008; doi:10.1371/journal.pone.0252390)
Supplement: S1 Table — (DOCX) [file pone.0252390.s004.docx]

| **Sample ID** | **Total Fragments** | **Unique Fragments** | **Unmapped Percent** | **Replicate Percent** | **Bridged Coverage (mode)** | **Base Coverage (mode)** | **Read Length (BP)** | **Junctions Reported** | **Tumor Percentage Determined by Mate Pair Sequencing** |
| --- | --- | --- | --- | --- | --- | --- | --- | --- | --- |
| **CTE003** | 102,955,814 | 95,469,080 | 0.92 | 6.35 | 52 | 3 | 151 | 325 | 97 |
| **CTE005** | 98,072,346 | 91,326,012 | 1.03 | 5.85 | 74 | 3 | 151 | 6 | 78 |
| **CTE006** | 91,315,187 | 84,523,216 | 1.01 | 6.43 | 72 | 3 | 151 | 198 | 89 |
| **CTE016** | 81,917,189 | 76,208,654 | 1.3 | 5.67 | 52 | 3 | 151 | 10 | 35 |
| **CTE024** | 85,282,063 | 79,183,302 | 1.34 | 5.81 | 56 | 3 | 151 | 238 | 49 |
| **CTE025** | 78,510,229 | 74,366,713 | 1.25 | 4.02 | 49 | 3 | 151 | 53 | 45 |
| **CTE029** | 99,392,603 | 94,007,913 | 1.33 | 4.08 | 62 | 3 | 151 | 327 | 40 |
| **CTE031** | 67,562,522 | 62,446,159 | 1.34 | 6.23 | 50 | 2 | 151 | 58 | 30 |
| **CTE033** | 93,561,724 | 85,660,489 | 0.82 | 7.62 | 60 | 3 | 151 | 274 | 50 |
| **CTE034** | 83,524,420 | 77,804,687 | 0.65 | 6.2 | 53 | 3 | 151 | 194 | 66 |
| **CTE037** | 68,864,621 | 64,956,114 | 0.74 | 4.94 | 35 | 2 | 151 | 233 | 88 |
|  |  |  |  |  |  |  |  |  |  |
| **Average** | 86,450,793 | 80,541,122 | 1.07 | 5.75 | 56 | 3 | 151 | 174 | 61 |

**S1 Table. Mate-pair sequencing quality control data.**
