# Supplementary material for: Personalized tumor-specific DNA junctions to detect circulating tumor in patients with endometrial cancer
Source: PLoS One. 2021 Jun 10;16(6):e0252390. doi: 10.1371/journal.pone.0252390 (PMC8192008; doi:10.1371/journal.pone.0252390)
Supplement: S8 Table — CHT, chemotherapy; EBRT, external beam radiation therapy; LND, lymphadenectomy; SLN, sentinel lymph node; VBT, vaginal brachytherapy. (DOCX) [file pone.0252390.s011.docx]

**S8 Table.** Patient demographics. CHT, chemotherapy; EBRT, external beam radiation therapy; LND, lymphadenectomy; SLN, sentinel lymph node; VBT, vaginal brachytherapy.

| **Clinical annotations** | **Number of patients (%)** |
| --- | --- |
| **Histology** |  |
| Serous | 5 (45.5 %) |
| Endometrioid | 3 (27.3 %) |
| Carcinosarcoma | 2 (18.2 %) |
| Mixed (Serous + Endometrioid) | 1 (9 %) |
| **Grade** |  |
| 1 | 1 (9.1 %) |
| 3 | 10 (90.9 %) |
| **Stage** |  |
| IA | 5 (45.5 %) |
| IIIA – IVB | 6 (54.5 %) |
| **Lymph node assessment** |  |
| SLN | 8 (72.7 %) |
| Pelvic LND | 3 (27.3 %) |
| **Adjuvant treatment** |  |
| No adjuvant treatment or only VBT | 2 (18.2%) |
| CHT (± VBT) | 5 (45.5 %) |
| CHT + EBRT | 4 (36.7 %) |
| **BMI** (median – IQR) | 36.6  (25.2 - 40.5) |
| **Age** (years, median - IQR) | 67  (65 - 71) |
| **Follow-up** (months from surgery, median - IQR) | 32  (27 - 36) |
